# Development and Psychometric Evaluation of the Scale of Externalizing Problem Behaviors in Adults (SEPBA): A Hybrid Dimensional–Categorical Instrument

**DOI:** 10.62641/aep.v54i2.2066

**Published:** 2026-04-15

**Authors:** Lidia Torres-Rosado, Óscar M. Lozano, Carmen Díaz-Batanero, Raúl Barba-Durán, Manuel Sanchez-Garcia

**Affiliations:** ^1^Department of Clinical and Experimental Psychology, University of Huelva, 21071 Huelva, Spain; ^2^Research Center on Natural Resources, Health and Environment, University of Huelva, 21071 Huelva, Spain; ^3^Huelva Penitentiary Center, General Secretariat of Penitentiary Institutions, 21610 Huelva, Spain

**Keywords:** externalizing behavior, psychometrics, statistical models, diagnosis, validation

## Abstract

**Background:**

The Scale of Externalizing Problem Behaviors in Adults (SEPBA) was developed as a hybrid psychometric instrument designed to assess both dimensional traits and categorical diagnoses associated with externalizing psychopathology. Based on an integrative operational framework drawing on *Diagnostic and Statistical Manual of Mental Disorders* (DSM-5) and the Hierarchical Taxonomy of Psychopathology (HiTOP) model, the SEPBA assesses 15 traits/facets and 6 disorders within the domains of antagonism and disinhibition.

**Methods:**

The present study analyzed the psychometric properties of the SEPBA in a combined community and prisoner sample (n = 922). The final version of the SEPBA included 144 items rated on a 4-point Likert scale. Reliability (α, ω), item discrimination, convergent/discriminant validity, confirmatory factor analyses, and gender measurement invariance were examined.

**Results:**

The results indicated adequate item discrimination and internal consistency across all scales. Confirmatory factor analyses supported the unidimensionality of the individual scales and the hypothesized hierarchical organization of traits and facets. Gender invariance of the measure was demonstrated. In addition, evidence of convergent and discriminant validity was provided through correlations with external measures.

**Conclusion:**

The findings support the SEPBA as a suitable assessment instrument. Its hybrid structure offers an integrative approach to transdiagnostic assessment, enhancing both the clinical utility of categorical diagnoses and the empirical applicability of dimensional profiles for assessing externalizing behaviors in adults.

## Introduction

Maladaptive externalizing behaviors are associated with interpersonal conflicts and difficulties in the individual’s social environment [1, 2, 3]. These behaviors are incorporated into diagnostic categories (e.g., antisocial, borderline, and attention-deficit/hyperactivity disorders) and are categorically assessed—indicating the presence or absence of a disorder—in the traditional nosotaxies of the *Diagnostic and Statistical Manual of Mental Disorders *(DSM) and the *International Classification of Diseases *(ICD). However, recent models such as the Hierarchical Taxonomy of Psychopathology (HiTOP), the Alternative Model for Personality Disorders (AMPD) from DSM-5 [4], and the latest version of the ICD [5], together with empirical evidence from other authors [6], advocate for the assessment of symptoms and traits along a dimensional continuum of severity. Most maladaptive externalizing behaviors fall primarily (though not exclusively) into two domains: *disinhibited externalizing*, associated with problematic impulsive traits; and *antagonistic externalizing*, which includes traits such as lack of empathy, conflict-seeking, or disregard for others’ feelings and rights [7, 8].

The suitability of categorical versus dimensional assessment of traits, symptoms, and disorders remains a central nosological debate, with significant implications for the diagnosis, treatment, and research of mental disorders [6, 9, 10]. In the field of personality disorders, practitioner consensus increasingly favors a hybrid categorical–dimensional system, whereby dimensional assessment captures the severity of personality functioning and traits that can be integrated to generate diagnoses [10]. Nevertheless, few psychometric instruments successfully integrate both types of assessments, largely due to the conceptual and methodological complexities of developing such hybrid systems. Currently, one example of a hybrid system is found in versions of the Structured Clinical Interview for DSM-5 [11]. However, the Structured Clinical Interview for DSM-5 (SCID-5) is a structured clinical interview that requires extensive training to administer, which limits its use by untrained professionals in clinical screening settings (e.g., primary care) and within research contexts. In such settings, dimensionally scored tests and scales are more commonly employed, relying on statistical cutoffs to establish a diagnostic threshold [12]. However, these thresholds are often based on post hoc statistical criteria rather than on clinical standards derived from formal diagnostic classifications (DSM or ICD). Consequently, applying such cutoffs in samples different from those in which they were estimated may lead to inaccuracies, including false positives or false negatives.

Several empirical studies [13, 14] and systematic reviews and meta-analyses [15, 16, 17, 18] have quantitatively examined the degree of correspondence between categorical and dimensional scores, generally reporting low-to-moderate effect sizes. The unshared statistical variance observed in these studies may have clinically meaningful implications. For instance, Hines *et al*. [19] analyzed a sample of patients diagnosed with borderline personality disorder using DSM-5 Section II criteria. When these patients were assessed using the AMPD framework, diagnostic convergence was observed in only 66.2% of cases, indicating that 33.8% of patients received non-overlapping diagnoses—potentially affecting treatment decisions.

Discrepancies between categorical and dimensional assessments may arise from conceptual differences in disorder definitions, measurement instruments, or sample characteristics, among other factors. Despite the complexity of integrating both evaluation systems into a hybrid model, advancing such integration remains a priority [20, 21]. In this regard, Torres-Rosado *et al*. [22] developed an operational definition that conceptually aligns diagnostic criteria from DSM-5 Section II with facets and traits defined in DSM-5 Section III, HiTOP, and the proposal by Mullins-Sweatt *et al*. [23]. This operational definition served as the basis for developing the *Scale of Externalizing Problem Behaviors in Adults* (SEPBA), designed as a hybrid instrument. Based on this premise, the present study aims to analyze the psychometric properties of the SEPBA by providing: (1) item-level metric properties for each scale (facets/traits and disorders); (2) reliability evidence (internal consistency) for facet/trait and disorder scores; (3) validity evidence based on the internal structure of each scale; (4) validity evidence based on the hierarchical structure of the externalizing spectrum as conceptualized in the HiTOP model; and (5) validity evidence based on the associations between SEPBA scores and external variables.

## Methods

### Participants and Procedure

The study sample consisted of 922 participants drawn from two subpopulations. Given that substantial heterogeneity is desirable for testing the psychometric properties of assessment instruments, the authors opted to use a community sample representative of the Spanish population and a sample drawn from an incarcerated population, a context in which maladaptive behaviors are more prevalent. To participate in the study, respondents were required to meet the following inclusion criteria: (i) be between 18 and 70 years old; (ii) be able to read and write; (iii) provide informed consent. Participants with any medical or psychological diagnosis that would preclude test administration were excluded.

The first subpopulation included 773 participants selected through stratified random sampling (by age group—*M* = 48.33; *SD* = 16.44—and gender—50.1% male and 49.9% female; other sociodemographic characteristics are reported in **Supplementary Table 1**) from the general population residing in Spain. Data were collected from January 8 to 29, 2024. The questionnaire was sent to 815 participants. Of these, 18 were excluded for providing contradictory responses to control questions, 6 for excessive response time, and 18 for incomplete questionnaires. Therefore, the response rate was 94.85%. The assessment instruments were administered online via a specialized company certified by the International Organization for Standardization (UNE-ISO 20252:2019). Data collection was conducted over two sessions, each lasting an average of 19.06 minutes (*SD* = 46.09). Control items were included to detect inattentive responding. Participants who completed the assessment in less than 20% of the average response time were excluded from the study.

The second subpopulation included 149 incarcerated individuals (*M* age = 41.67; *SD* = 11.06; gender: 88.6% male, 11.4% female) serving criminal sentences in a prison. This subsample was recruited via convenience sampling, whereby prison staff selected participants based on availability. Assessment for this subsample was conducted individually by a psychologist from the research team in a private room within the facility from February 1 to June 30, 2024. Before the assessment, participants were informed of the study objectives, the confidentiality of their responses, and that their responses would not be seen by prison staff or included in their files. It was also made clear that participation was voluntary and would not result in any penitentiary benefits. Participants provided written informed consent before enrolling. Each interview lasted approximately 45 minutes.

The study was conducted in accordance with the Declaration of Helsinki and was approved by the Biomedical Research Ethics Committee of Andalucia (approval code: 0317-N-22). All participants provided written informed consent prior to participation.

## Measures

### Scale of Externalizing Problem Behaviors in Adults (SEPBA)

The SEPBA was developed based on the operational definition described in Torres-Rosado *et al*. [22]. This definition integrates diagnostic criteria and facets/traits from DSM-5 and from the proposal by Mullins-Sweatt *et al*. [23], both related to the domains of disinhibition and antagonism. It also includes internalizing traits to assess criteria specific to borderline personality disorder. Overall, 66 diagnostic criteria from seven DSM-5 disorders were mapped onto 15 facets (the SEPBA does not include oppositional defiant disorder).

Initially, 10 to 15 items were written for each diagnostic criterion, following the guidelines of the Standards for Educational and Psychological Testing [24]. These items were revised by two psychometric experts from the research team, who selected between 6 and 8 items for each DSM diagnostic criterion. The selected items were grouped into evaluation packages and sent to 38 external experts, ensuring that each item was reviewed by at least five of the experts. Items were rated for relevance in measuring the intended content using a 5-point scale (1 = low relevance, 5 = high relevance). The Content Validity Index (CVI) [25], Content Validity Ratio (CVR) [26], and Aiken’s V index (Aiken [27]), were calculated, with a threshold of ≥0.70 used to determine item acceptability. For each diagnostic criterion, the four items with the highest validity indices were retained and included in a preliminary version consisting of 576 items. This pilot version was administered to a sample of 364 participants recruited through convenience sampling from the general community and from treatment centers for individuals diagnosed with conduct-related disorders (e.g., mental health or addiction services). Item analyses assessed the discrimination index, floor and ceiling effects, and unidimensionality for both facets/traits and disorders. Based on these analyses, two items per criterion with the strongest psychometric properties were selected.

The final SEPBA instrument (**Supplementary Table 2**) comprises 144 items covering the antagonism and disinhibition domains, and includes internalizing facets relevant to borderline and histrionic personality disorder. Of these, a total of 122 items assess 15 facets/traits: rule/law violations (6 items), impulsivity (8), physical aggression (6), risk-taking (4), irresponsibility (6), inattention (18), hyperactivity (12), lack of rigid perfectionism (6), deceitfulness (6), lack of empathy (6), attention-seeking (10), grandiosity (12), exploitative (6), suspiciousness (10), and hostility (6). A total of 116 items assess six personality disorders: antisocial (14 items, 7 diagnostic criteria), histrionic (16 items, 8 criteria), narcissistic (18 items, 9 criteria), paranoid (14 items, 7 criteria), borderline (18 items, 9 criteria), and ADHD (36 items: 18 for inattention and 18 for hyperactivity/impulsivity). An additional 22 items assess clinically relevant content from the internalizing spectrum corresponding to borderline and histrionic personality disorder, in accordance with DSM-5 and HiTOP. **Supplementary Table 3** provides the test specification table, including the item numbers assessing each facet/trait and each diagnostic disorder.

Items were scored using a 4-point Likert scale (1 = strongly disagree, 4 = strongly agree).

The SEPBA provides two types of scores: dimensional and categorical. The dimensional score evaluates the facets/traits and is obtained by calculating the mean of the items comprising each facet (**Supplementary Table 2**), with scores ranging from 1 to 4. Higher scores indicate greater severity of the assessed facet/trait.

The categorical score is used to identify disorders. For this purpose, the two items that operationalize each diagnostic criterion are summed (**Supplementary Table 2**), yielding a score ranging from 2 to 8. Following the cutoffs provided in the results section, a value of ‘0’ is assigned when the score is below the cutoff, and a score of ‘1’ is assigned when the value meets or exceeds the cutoff. As a result, each diagnostic criterion is evaluated as ‘0’ (absent) or ‘1’ (present). Finally, the diagnostic criteria for each disorder are summed to establish disorder presence according to the DSM-5.

### Instruments for Convergent and Discriminant Validity Evidence

To examine the convergent validity of SEPBA facets/traits and disorders, the following Spanish-language instruments were administered: the Externalizing Spectrum Inventory [28], the Personality Inventory for DSM-5 Short Form [29], the International Personality Disorder Examination Screening Questionnaire -IPDEQ- [30], and the Adult ADHD Self-Report Scale v1.1 [31].** Supplementary Table 4** provides a list of the instruments, associated subscales, and their reliability coefficients within the study sample.

## Analysis

Item discrimination was assessed using corrected item-total correlations, with values ≥0.30 considered indicative of acceptable discrimination [32]. To analyze item validity evidence, correlations were computed between SEPBA items and theoretically related external scales, with values ≥0.25 considered adequate [32].

Internal consistency reliability for each SEPBA scale was estimated using both Cronbach’s alpha and McDonald’s omega coefficients. Following recommendations for clinical and research instruments, coefficients ≥0.80 were considered acceptable [33].

Confirmatory factor analysis (CFA) was used to assess the unidimensionality of each SEPBA scale. The estimation method employed was diagonally weighted least squares (DWLS), implemented via the cfa-function in the lavaan R package [34]. This method is appropriate for ordinal data when multivariate normality cannot be assumed [35, 36].

Additionally, a hierarchical CFA model was used to test validity evidence based on the structure of the externalizing spectrum as conceptualized in the HiTOP model. The tested model included the 15 SEPBA trait/facet scales grouped into two first-order domains—antagonism and disinhibition—which, in turn, loaded onto a higher-order externalizing factor. As noted by Bollen [37], second-order CFAs typically require at least three first-order factors for model identification. However, the hierarchical HiTOP model tested here includes only two first-order factors (antagonism and disinhibition), posing an identification issue. This issue was addressed following the recommendation of Rossen *et al*. [38] to equate the factor loadings from the second-order factor onto the first-order factors.

Model fit was evaluated using several indices: the Comparative Fit Index (CFI), Tucker–Lewis Index (TLI), and Root Mean Square Error of Approximation (RMSEA). Good fit was defined as CFI and TLI >0.95 and RMSEA <0.08.

Gender-based measurement invariance for the hierarchical CFA model was tested in four steps: configural, metric, scalar, and strict invariance. Model comparisons were conducted between increasingly constrained models. Invariance was assumed when changes in CFI and RMSEA were less than 0.01 and 0.015, respectively [39, 40].

For convergent and discriminant validity evidence, correlations between SEPBA trait and disorder scores and theoretically related external scales were expected to be at least r >0.50 [41]. Correlations between SEPBA scores and theoretically related facets/traits and diagnoses were examined to ensure that they were significantly stronger than correlations with theoretically unrelated constructs [42]. Finally, cutoff points for determining the presence of disorders evaluated by the SEPBA were estimated using ROC analysis, with the cutoff determined based on Youden’s index.

All analyses were conducted using R [43] and SPSS.29.0.1 [44] software.

## Results

### Item-level Analysis and Reliability Estimation of Facets/Traits and Disorders

Table 1 presents the item analysis for the 15 SEPBA facets/traits evaluated. Discrimination indices were adequate according to the criteria adopted, with the lowest corrected item-total correlation observed for an item from the suspiciousness scale (*r* = 0.33). Item validity analyses showed that most items achieved moderate to high correlations with external instruments measuring similar content. Nonetheless, a subset of items on certain scales exhibited relatively weak associations, most notably within the *rigid perfectionism* trait, whose items yielded the lowest validity coefficients when compared with scores on the corresponding Personality Inventory for DSM-5 (PID-5) scale. Internal consistency, estimated using both Cronbach’s alpha and McDonald’s omega, was found to be satisfactory.

**Table 1.** **Discrimination and validity indices of items and internal consistency of the SEPBA, organized by facets/traits**.

Facets/traits	Nº of items	Discrimination index		Item validity index				Internal consistency	
		Min.	Max.	Facets/Traits (Scale)	Min.	Max.	Number of items with r < 0.25	α	ω
Rule/law violations	6	0.41	0.66	Rebelliousness (ESI)	0.19	0.47	1	0.80	0.81
				Physical Aggression (ESI)	0.25	0.43	0		
Impulsivity	8	0.43	0.59	Impulsivity (PID-100)	0.19	0.47	1	0.81	0.81
				Impulsivity (ASRS)	0.13	0.50	2		
Physical aggression	6	0.53	0.74	Rebelliousness (ESI)	0.29	0.46	0	0.87	0.87
				Physical Aggression (ESI)	0.34	0.55	0		
Risk-taking	4	0.51	0.73	Risk Taking (PID)	0.40	0.61	0	0.83	0.84
Irresponsibility	6	0.42	0.59	Irresponsibility (PID)	0.31	0.45	0	0.77	0.78
Inattention	18	0.55	0.75	Inattention (ASRS)	0.39	0.53	0	0.94	0.94
Hyperactivity	12	0.50	0.80	Hyperactivity (ASRS)	0.25	0.52	0	0.93	0.93
Lack of rigid perfectionism	6	0.42	0.68	Irresponsibility (PID)	0.18	0.41	1	0.81	0.82
				Distractibility (PID)	0.22	0.42	1		
				Lack of Rigid Perfectionism (PID)	–0.17	0.09	6		
Deceitfulness	6	0.51	0.74	Deceitfulness (PID)	0.37	0.57	0	0.87	0.87
Lack of empathy	6	0.45	0.69	Callousness (PID)	0.25	0.41	0	0.83	0.83
Attention seeking	10	0.40	0.72	Attention Seeking (PID)	0.20	0.57	1	0.89	0.89
Grandiosity	12	0.43	0.60	Grandiosity (PID)	0.22	0.45	3	0.85	0.86
Exploitative	6	0.43	0.61	Manipulativeness (PID)	0.22	0.56	2	0.80	0.79
Suspiciousness	10	0.33	0.69	Suspiciousness (PID)	0.20	0.54	1	0.87	0.87
Hostility	6	0.43	0.69	Hostility (PID)	0.29	0.56	0	0.83	0.83

*Note*: SEPBA, Scale of Externalizing Problem Behaviors in Adults; ESI, Externalizing Spectrum Inventory; PID, Personality Inventory for DSM-5; ASRS, Adult ADHD Self-Report Scale. Discrimination index = corrected item–total correlations.

Table 2 presents the item analysis organized by diagnostic categories. Discrimination indices and most item validity values were acceptable across disorders. The only exception was the *Histrionic Personality Disorder* Scale, for which 5 out of 16 items showed correlations below 0.25 with external variables. Estimates of internal consistency were also satisfactory for all disorders.

**Table 2.** **Discrimination and validity indices of items and internal consistency of the SEPBA, organized by disorders**.

Disorder	Nº of items	Discrimination index		Item validity index				Internal consistency	
		Min.	Max.	Disorder	Min.	Max.	Number of items with r < 0.25	α	ω
Antisocial	14	0.41	0.67	Antisocial (IPDE)	0.20	0.61	2	0.89	0.89
Histrionic	16	0.35	0.63	Histrionic (IPDE)	0.19	0.40	5	0.87	0.87
Narcissistic	18	0.43	0.63	Narcissistic (IPDE)	0.19	0.39	2	0.89	0.89
Paranoid	14	0.38	0.66	Paranoid (IPDE)	0.19	0.47	1	0.89	0.89
Borderline	18	0.41	0.70	Borderline (IPDE)	0.33	0.53	0	0.91	0.91
Inattention	18	0.55	0.75	Inattention (ASRS)	0.38	0.53	0	0.94	0.94
Hyperactivity	18	0.50	0.75	Hyperactivity (ASRS)	0.29	0.48	0	0.93	0.93

*Note*: IPDE, International Personality Disorder Examination Screening Questionnaire; ASRS, Adult ADHD Self-Report Scale. Discrimination index = corrected item–total correlations.

### Validity Evidence Based on Internal Structure of Facets/Traits and Disorders

Table 3 presents the model fit indices for unidimensional CFA models for each facet/trait and disorder. CFI and TLI values were acceptable for all facet/trait scales except *impulsivity*, which exhibited marginal fit indices. RMSEA values exceeded the recommended threshold in 8 of the 15 trait scales. A detailed analysis of the residuals and modification indices largely explains these values: most items for the dimensional facets/traits correspond to items generated to measure each diagnostic criterion using two parallel items. The highest residual values and modification indices consistently corresponded to these paired items. On the other hand, it should be noted that, according to Lai and Green [45], it is not uncommon to find inconsistent CFI and RMSEA fit indices in factor analyses, which do not necessarily indicate model misspecification or flaws in the data. In all cases, factor loadings were equal to or greater than 0.40. Similarly, all items used to assess the disorders demonstrated acceptable fit to unidimensional CFA models and showed significant factor loadings (Table 3).

**Table 3.** **Fit indices for facets/traits and disorders**.

	Facets/traits	Nº of items	Unidimensionality				
			Fit Index			Factorial loadings	
			CFI	TLI	RMSEA	Min.	Max.
Facets/traits	Rule/law violations	6	0.99	0.99	0.065	0.55	0.83
	Impulsivity	8	0.92	0.88	0.259	0.58	0.81
	Physical aggression	6	0.99	0.99	0.028	0.70	0.89
	Risk-taking	4	0.99	0.99	0.000	0.64	0.85
	Irresponsibility	6	0.99	0.98	0.089	0.63	0.74
	Inattention	18	0.99	0.99	0.095	0.62	0.83
	Hyperactivity	12	0.99	0.99	0.093	0.66	0.89
	Lack of rigid perfectionism	6	0.99	0.99	0.000	0.55	0.85
	Deceitfulness	6	0.99	0.99	0.023	0.68	0.86
	Lack of empathy	6	0.99	0.99	0.012	0.59	0.86
	Attention seeking	10	0.99	0.98	0.115	0.56	0.88
	Grandiosity	12	0.96	0.95	0.126	0.52	0.78
	Exploitative	6	0.99	0.99	0.070	0.56	0.81
	Suspiciousness	10	0.99	0.98	0.086	0.41	0.82
	Hostility	6	0.98	0.96	0.180	0.54	0.85
Disorders	Antisocial	14	0.97	0.97	0.106	0.61	0.81
	Histrionic	16	0.94	0.93	0.150	0.40	0.85
	Narcissistic	18	0.96	0.96	0.104	0.51	0.77
	Paranoid	14	0.97	0.97	0.107	0.47	0.81
	Borderline	18	0.99	0.98	0.074	0.46	0.80
	Inattention	18	0.99	0.98	0.095	0.62	0.83
	Hyperactivity	18	0.97	0.96	0.159	0.66	0.87

*Note*: CFI, Comparative Fit Index; TLI, Tucker–Lewis Index; RMSEA, Root Mean Square Error of Approximation.

### Validity Evidence Based on the Hierarchical Structure of the Externalizing Spectrum

**Fig. 1.**
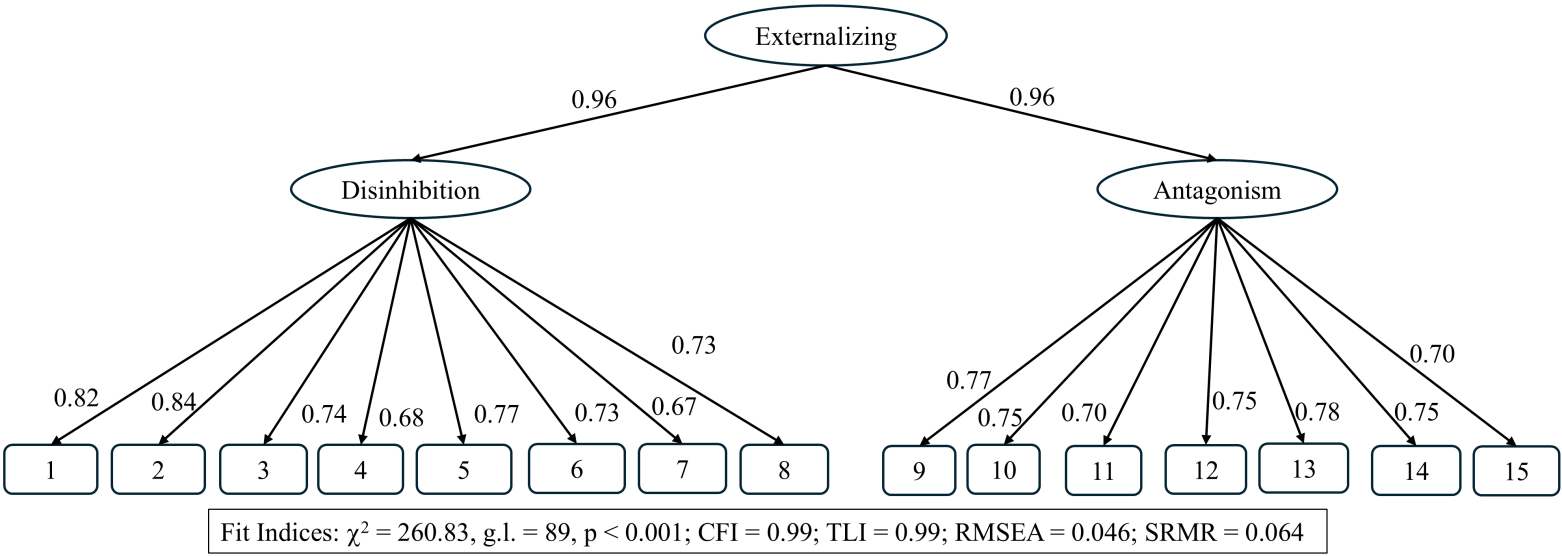
**Fit indices for the HiTOP disinhibition and antagonist domains**. *Note*: 1. Rules/law violations; 2. Impulsivity; 3. Physical aggression; 4. Risk-taking; 5. Irresponsibility; 6. Inattention; 7. Hyperactivity; 8. Lack of rigid perfectionism; 9. Deceitfulness; 10. Lack of empathy; 11. Attention seeking; 12. Grandiosity; 13. Exploitative; 14. Suspiciousness; 15. Hostility.

The CFA results assessing the hierarchical structure proposed by HiTOP are shown in Fig. 1. Model fit indices were adequate, and the factor loadings of the traits onto the domain-level factors were substantial. The high loadings from the second-order factor onto the first-order factors suggest potential unidimensionality. To test this assumption, two alternative CFA models were evaluated: a unidimensional model (15 facets, one dimension) and a two-correlated-factors model. Both models showed good fit (CFI and TLI = 0.99; RMSEA values of 0.049 and 0.046, and SRMR values of 0.067 and 0.064, respectively). Given the statistical equivalence of the models, the model that is more widely accepted and better supported in the scientific literature was retained (see Fig. 1).

Measurement invariance testing across gender groups (Table 4) showed adequate model fit at each step, confirming configural, metric, scalar, and strict invariance. These results indicate that the SEPBA maintains a stable factorial structure for both men and women.

**Table 4.** **Gender-based measurement invariance**.

Modelo	χ ^2^	df	CFI	ΔCFI	TLI	SRMR	RMSEA	ΔRMSEA
*Male*	271.47	89	0.980		0.976	0.081	0.063	
*Female*	53.41	89	0.999		0.999	0.050	0.000	
M1: Configural invariance	324.88	178	0.991		0.989	0.063	0.042	
M2: Metric invariance (M1 vs. M2)	408.52	194	0.987	0.004	0.985	0.073	0.049	0.008
M3: Scalar invariance (M2 vs. M3)	452.34	206	0.985	0.002	0.984	0.075	0.051	0.002
M4: Strict invariance (M3 vs. M4)	515.50	221	0.982	0.003	0.983	0.085	0.054	0.003

*Note*: Configural invariance = factor structure equal across groups; metric invariance = factor loadings constrained to equality; scalar invariance = intercepts constrained to equality; strict invariance = residuals constrained to equality.

### Convergent and Discriminant Validity Evidence

Table 5 displays the correlations between SEPBA facet/trait scores and corresponding scores from external instruments. Correlations for theoretically related constructs (convergent evidence) are shown in bold. These convergent correlations were generally moderate to high (*r* = 0.44–0.68), except for the *rigid perfectionism* facet from the PID-5. Regarding discriminant evidence, no correlation exceeded those observed for convergent validity. Of the 235 discriminant correlations tested, 225 were significantly lower than their corresponding convergent correlations.

**Table 5.** **Convergent and discriminant correlations between SEPBA facet/trait scores and external instruments**.

	Correlations																
	1	2	3	4	5	6	7	8	9	10	11	12	13	14	15	16	17
Rule/law violations	**0.57**	0.48	0.27	0.44	0.42	0.43	0.29	0.28	0.31	0.20	0.51	0.37	0.30	0.27	0.47	0.44	0.39
Impulsivity	0.37	0.34	**0.52**	**0.52**	0.37	0.49	0.38	0.39	0.42	0.18	0.47	0.28	0.38	0.27	0.39	0.42	0.43
Physical aggression	0.46	**0.59**	0.21	0.46	0.45	0.37	0.16	0.27	0.21	0.23	0.44	0.39	0.24	0.20	0.37	0.44	0.48
Risk-taking	0.51	0.56	0.19	0.55	**0.68**	0.46	0.13	0.31	0.22	0.32	0.41	0.29	0.36	0.12	0.42	0.54	0.35
Irresponsibility	0.40	0.43	0.23	0.49	0.40	**0.56**	0.30	0.29	0.34	0.18	0.42	0.25	0.30	0.13	0.34	0.46	0.35
Inattention	0.29	0.16	0.43	0.41	0.16	0.50	**0.66**	0.39	0.70	0.05	0.34	0.26	0.22	0.24	0.23	0.29	0.32
Hyperactivity	0.26	0.19	0.41	0.37	0.25	0.37	0.38	**0.55**	0.40	0.16	0.34	0.26	0.28	0.27	0.30	0.27	0.28
Lack of rigid perfectionism	0.31	0.17	0.32	0.34	0.13	**0.44**	**0.48**	0.27	**0.46**	–0.03	0.38	0.31	0.18	0.28	0.28	0.21	0.29
Deceitfulness	0.44	0.46	0.27	0.42	0.44	0.53	0.24	0.25	0.28	0.18	**0.63**	0.39	0.40	0.28	0.53	0.43	0.33
Lack of empathy	0.32	0.27	0.23	0.28	0.20	0.35	0.25	0.20	0.26	0.11	0.40	**0.48**	0.21	0.34	0.32	0.28	0.29
Attention seeking	0.28	0.18	0.32	0.25	0.22	0.34	0.27	0.28	0.26	0.17	0.43	0.22	**0.65**	0.37	0.42	0.22	0.23
Grandiosity	0.30	0.28	0.29	0.25	0.30	0.34	0.22	0.23	0.24	0.29	0.47	0.33	0.48	**0.50**	0.47	0.39	0.25
Exploitative	0.31	0.25	0.30	0.27	0.20	0.36	0.29	0.19	0.29	0.18	**0.53**	0.39	0.40	0.46	**0.47**	0.30	0.29
Suspiciousness	0.38	0.45	0.27	0.44	0.45	0.44	0.26	0.35	0.30	0.32	0.46	0.37	0.28	0.25	0.36	**0.68**	0.45
Hostility	0.40	0.44	0.27	0.51	0.39	0.38	0.23	0.36	0.31	0.34	0.41	0.33	0.26	0.23	0.37	0.51	**0.58**

*Note*: 1 = Rebelliousness (ESI); 2 = Physical aggression (ESI); 3 = Inattention (ASRS); 4 = Hyperactivity/Impulsivity (ASRS); 5 = Risk taking (PID); 6 = Irresponsibility (PID); 7 = Inattention (ASRS); 8 = Hyperactivity (ASRS); 9 = Distractibility (PID); 10 = Lack of rigid perfectionism (PID); 11 = Deceitfulness (PID); 12 = Callousness (PID); 13 = Attention seeking (PID); 14 = Grandiosity (PID); 15 = Manipulativeness (PID); 16 = Suspiciousness (PID); 17 = Hostility (PID). Bold values represent the highest correlations for each facet/trait.

Similarly, Table 6 presents the convergent correlations between SEPBA-assessed personality disorders and those assessed by the IPDEQ, which are shown in bold. All convergent correlations were significantly greater than the corresponding discriminant correlations, except for the correlation between paranoid personality disorder (SEPBA) and borderline personality disorder (IPDEQ).

**Table 6.** **Convergent and discriminant correlations between SEPBA facet/trait scores and external instruments**.

Disorders	Correlations						
	Antisocial (IPDE)	Histrionic (IPDE)	Narcissistic (IPDE)	Paranoid (IPDE)	Borderline (IPDE)	Inattention (ASRS)	Hyperactivity (ASRS)
Antisocial	**0.66**	0.38	0.35	0.44	0.51	0.20	0.31
Histrionic	0.33	**0.50**	0.40	0.26	0.46	0.37	0.42
Narcissistic	0.29	0.34	**0.53**	0.24	0.34	0.26	0.30
Paranoid	0.49	0.30	0.32	**0.58**	0.55	0.27	0.39
Borderline	0.49	0.42	0.29	0.44	**0.65**	0.37	0.44
Inattention	0.20	0.26	0.16	0.15	0.38	**0.66**	0.45
Hyperactivity	0.25	0.35	0.26	0.19	0.37	0.43	**0.59**

*Note*: IPDE, International Personality Disorder Examination Screening Questionnaire; ASRS, Adult ADHD Self-Report Scale. Bold values represent convergent correlations.

Table 7 shows the cutoffs and indicators of the clinical utility of the SEPBA. Columns C1-C9 display the diagnostic criteria for each disorder, ordered as they appear in the DSM-5. Each cell contains the cutoff value for the corresponding diagnostic criterion, derived from the sum of the two items that operationalize it (**Supplementary Table 2**). Indicators of clinical utility are then presented, using the IPDEQ (for convergent validity) or the ASRS as the gold standard, as appropriate. Overall, all disorders evaluated by the SEPBA showed agreement levels above 75%, with moderate kappa indices for all disorders except the histrionic and narcissistic scales. Regarding sensitivity and specificity, the reported values indicate that the use of these cutoffs increases specificity at the expense of sensitivity.

**Table 7.** **Cutoffs for DSM-5 criteria and clinical utility estimates for the SEPBA**.

Disorder	Cutoffs for diagnostic criteria									Disorder	Clinical utility statistics			
SEPBA scales	C1	C2	C3	C4	C5	C6	C7	C8	C9	Gold standard	% agreement	Kappa	Sensitivity	Specificity
Antisocial	4	5	5	4	5	5	5			Antisocial (IPDE)	83%	0.51	0.78	0.84
Histrionic	5	5	5	6	5	4	6	5		Histrionic (IPDE)	86%	0.30	0.36	0.93
Narcissistic	5	5	5	5	4	5	4	5	5	Narcissistic (IPDE)	84%	0.38	0.64	0.86
Paranoid	5	5	5	5	5	6	5			Paranoid (IPDE)	77%	0.44	0.66	0.81
Inattention	5	5	5	5	5	5	4	5	5	Inattention (ASRS)	85%	0.49	0.83	0.86
Hyperactivity and impulsivity	5	5	5	5	5	5	6	4	5	Hyperactivity and impulsivity (ASRS)	85%	0.42	0.54	0.90

## Discussion

The SEPBA was designed to provide both dimensional scores based on facets/traits within the *antagonism* and *disinhibition *domains, and categorical DSM-5 diagnoses, thereby serving as a hybrid psychometric instrument aligned with the needs of both clinicians and researchers [10]. The authors consider that the SEPBA is theoretically grounded by the operational definition process followed. Furthermore, the empirical evidence from this study supports the internal consistency of the dimensional and categorical scales, as well as the validity of their internal structure and their convergent and discriminant relationships with similar constructs. Likewise, preliminary evidence has been provided regarding its clinical utility, although this aspect warrants further study using specific clinical samples.

Item-level analyses demonstrated adequate discrimination indices for both the dimensional assessment of facets/traits and the categorical diagnosis of disorders. This finding is particularly relevant for the hybrid approach proposed in the SEPBA, as it not only enables precise differentiation in severity levels across traits but also supports DSM-5-aligned diagnostic profiling. Furthermore, the observed item validity indices—except for the *rigid perfectionism* facet in the PID-5—based on correlations with external instruments that measure similar traits and disorders, further support SEPBA’s transdiagnostic utility in clinical settings [46].

With regard to internal consistency, values above 0.80 are recommended for instruments used in diagnostic or clinical decision-making contexts [47], a criterion met by most SEPBA scales. Achieving such reliability helps reduce Type I and Type II diagnostic errors by minimizing measurement error. High internal consistency is also important for interpreting individual-level scores, particularly when assessing clinically significant change. Notably, the high internal consistency observed for both facets/trait and disorder scales does not appear to result from item redundancy—a common source of inflated reliability [48]—as the items were derived from distinct diagnostic criteria. Taken together, the SEPBA yields scores that are both psychometrically and clinically meaningful.

The present results also highlight evidence of SEPBA’s convergent and discriminant validity, which is particularly relevant given the high comorbidity typically observed in mental disorder assessments [49]. While such comorbidity is common, instruments must nevertheless demonstrate sufficient discriminant capacity. In this regard, the SEPBA not only demonstrated strong convergent validity but also distinguished the specific features of each disorder, thereby enabling differential diagnosis [50]. Together with its unidimensional scale structure and replication of HiTOP’s conceptual hierarchy [7], these findings suggest that the high comorbidity observed with the SEPBA should not be interpreted as diagnostic overlap but rather as the expression of latent traits shared across disorders, as conceptualized in models such as HiTOP or AMPD. Clinically, this hybrid approach enables precise monitoring of traits that distinguish disorders while avoiding the masking of shared traits that could lead to diagnostic errors. Moreover, it facilitates the characterization of psychopathology within dimensional assessments [51]. From a research perspective, precise trait discrimination supports etiological studies aimed at identifying endophenotypes [52] and disorder-specific biomarkers [53].

In addition to structural and relational validity, the present study provides preliminary indicators of clinical utility. Using the IPDEQ and the ASRS as reference standards, the SEPBA achieved agreement rates above 75% across disorders, with moderate kappa values except for the histrionic and narcissistic scales. These results suggest that the SEPBA can serve as a screening tool; however, caution is warranted. Identifying optimal cutoffs is inherently complex because sensitivity and specificity vary with the threshold and the gold standard used. In our analyses, the proposed cutoffs prioritize specificity over sensitivity, which may reduce false positives but increase false negatives—a trade-off that must be considered in clinical decision-making [54]. Furthermore, the IPDEQ has limitations, as its validity and reliability have been debated, particularly in forensic samples [55]. It is a brief screening questionnaire rather than a diagnostic gold standard. As such, its associations with the SEPBA should be interpreted as evidence of convergent validity rather than diagnostic-level criterion validity. This distinction is intended to underscore the utility of screening instruments as efficient indices of liability to personality pathology, ideally complemented by semi-structured diagnostic interviews (e.g., SCID-5, IPDE interview). Therefore, future research should establish SEPBA cutoffs against structured clinical interviews in well-characterized clinical samples. Such studies would allow the estimation of sensitivity, specificity, and predictive values under conditions that reflect real-world diagnostic complexity, thereby improving the interpretability and generalizability of SEPBA’s categorical thresholds.

Although the present findings support the utility of hybrid SEPBA scores, some limitations should be acknowledged. On the one hand, although the community sample is representative of the general population, the prison sample was recruited through convenience sampling. This sample was selected by prison professionals from among volunteers, with an emphasis on incarcerated individuals who exhibited respectful behavior toward others. As a result, a selection bias may have been introduced in this subsample, which could affect the external validity of the findings.

Psychometrically, a key issue was the lack of convergent validity between the SEPBA *rigid perfectionism* scale and the equivalent scale in the PID-100, despite both targeting the same construct. This discrepancy led the authors to perform additional analyses using theoretically related scales. The low correlations between the SEPBA and PID-5 rigid perfectionism scales may reflect methodological factors. Specifically, the PID-5 facet includes negatively worded items, which can introduce method variance and thus attenuate convergent validity. On the other hand, unidimensional CFA analyses showed that some scales yielded elevated RMSEA values, requiring further inspection and cautious interpretation. However, discrepancies between fit indices such as CFI and RMSEA are not uncommon and do not necessarily indicate model misspecification [45], as these indices assess fit from different perspectives and their cutoff values are somewhat arbitrary.

Nevertheless, future research should further examine the appropriateness of empirically derived thresholds.

## Conclusion

Despite these limitations, the present findings suggest that the SEPBA is a hybrid instrument with strong psychometric properties that support its use in both clinical contexts—particularly those with a transdiagnostic orientation—and research settings.

## Availability of Data and Materials

The datasets and materials generated and analyzed during the current study are openly available in the Open Science Framework (OSF) at https://osf.io/4uqzh/.
