# Supplementary material for: Development and Psychometric Evaluation of the Scale of Externalizing Problem Behaviors in Adults (SEPBA): A Hybrid Dimensional–Categorical Instrument
Source: Actas Esp Psiquiatr. 2026 Apr 15;54(2):335–47. doi: 10.62641/aep.v54i2.2066 (PMC13180669; doi:10.62641/aep.v54i2.2066)
Supplement: Supplementary file 1 [file ActEsp-54-2-335-347-s1.zip › AEP-2066-Supplement material.docx]

Supplementary Table 1. Sociodemographic characteristics of the community sample

|  |  | ***M* (*SD*)** |
| --- | --- | --- |
| Age |  | 47.26 (15.88) |
|  | **Frequency (%)** |  |
| Gender | |  |
| Men | 519 (56.3%) |  |
| Women | 130 (43.7%) |  |
| Marital status | |  |
| Single | 267 (29.0%) |  |
| Married or living with partner | 417 (45.2%) |  |
| Separated or divorced | 61 (6.6%) |  |
| Widowed | 28 (3.0%) |  |
| Educational level | |  |
| No formal education | 9 (1.0%) |  |
| Primary education | 15 (1.6%) |  |
| Secondary education | 361 (46.7%) |  |
| University education | 388 (50.2%) |  |
| Employment status | |  |
| Employed | 452 (58.5%) |  |
| Retired/pensioned | 171 (22.1%) |  |
| Unemployed | 102 (13.2%) |  |
| Student | 48 (5.2%) |  |
| Living arrangement |  |  |
| Living alone | 98 (12.6%) |  |
| Living with partner and/or child | 516 (66.8%) |  |
| Living with non-family members | 18 (2.33%) |  |
| Other situations | 15 (1.9%) |  |

Supplementary Table 2. Items of the final version of the SEPBA (Spanish)

| 1. Me gusta atraer la atención sobre mí mismo/a |
| --- |
| 2. Tengo cambios de humor constantes |
| 3. Cuando hablo me gusta utilizar expresiones elocuentes más que aportar detalles |
| 4. Las percepciones de otros terminan cambiando mi propia opinión |
| 5. Siento que las relaciones que tengo con los demás son más importantes para mí de lo que son para ellos |
| 6. Me considero una persona muy importante |
| 7. No me importa que los demás trabajen para mí |
| 8. Pienso que los demás intentan hacerme daño |
| 9. Si alguien me insulta, no lo olvido |
| 10. Tengo ataques de ira cuando alguien me abandona |
| 11. Mis relaciones personales son inestables |
| 12. Ante situaciones de estrés me vuelvo muy inestable emocionalmente |
| 13. Soy muy cambiante, incluso en mis valores personales |
| 14. He intentado quitarme la vida en más de una ocasión |
| 15. Siento como si no tuviera ningún propósito en la vida |
| 16. Pierdo el contacto con la realidad en momentos de estrés |
| 17. Cometo errores por no prestar suficiente atención |
| 18. No puedo parar de mover las manos y/o pies cuando estoy sentado/a |
| 19. No me importa cometer errores si puedo acabar pronto |
| 20. En mis tareas diarias me cuesta prestar atención a los detalles |
| 21. He intentado llevarme cosas que no me pertenecen |
| 22. No me ha importado mentir para conseguir lo que quiero |
| 23. En mi vida profesional he tomado decisiones sin pensar en las consecuencias |
| 24. Soy agresivo/a con otros |
| 25. Me he expuesto a situaciones peligrosas por mi imprudencia |
| 26. Por mi irresponsabilidad, he contraído deudas económicas |
| 27. No me preocupan las consecuencias de mis actos sobre los demás |
| 28. Necesito que la gente se fije en mí |
| 29. Mis emociones son inestables |
| 30. Soy vehemente hablando, aunque no suelo aportar argumentos |
| 31. Las opiniones de los demás influyen mucho en mí |
| 32. Considero amigos/as a personas que apenas conozco |
| 33. A los demás les cuesta reconocer mi gran valía |
| 34. Los demás deben ayudarme, aunque les suponga un inconveniente |
| 35. Desconfío de la gente que me rodea |
| 36. Me cuesta perdonar a quien me hace daño |
| 37. Haría lo que fuese por evitar que alguien me abandone |
| 38. Una persona que ayer adoraba hoy puede ser mi enemigo |
| 39. Tengo cambios de humor intensos que afectan a mi vida diaria |
| 40. La imagen que tengo de mí mismo/a cambia constantemente |
| 41. Me hago daño como forma de aliviar mi angustia |
| 42. Siento una sensación de vacío en mi interior |
| 43. En situaciones de tensión, pierdo la conexión con la realidad |
| 44. Tengo dificultades para mantener la atención |
| 45. Me muevo constantemente en mi asiento |
| 46. No me preocupa el cumplimiento de normas, siempre que el trabajo esté hecho |
| 47. Me resulta difícil mantener la atención en actividades recreativas |
| 48. He acosado a compañeros/as |
| 49. He falsificado documentos para beneficio propio |
| 50. En mi vida personal he tomado decisiones sin pensar en las consecuencias |
| 51. Ejerzo violencia física sobre otras personas |
| 52. He asumido riesgos que han puesto en peligro a otras personas |
| 53. Me han expulsado de algunos trabajos por irresponsable |
| 54. Aunque haya hecho daño a otras personas, no siento remordimiento |
| 55. Me gusta coquetear con la gente que me rodea |
| 56. Anhelo tener gran poder e influencia |
| 57. No me preocupa el esfuerzo que tengan que realizar otros, mientras que sepan cubrir mis necesidades |
| 58. Pienso que mis amigos/as no son totalmente honestos/as |
| 59. Contesto con ira cuando los demás me atacan |
| 60. Olvido cosas importantes en mis actividades diarias |
| 61. Tengo dificultades para mantenerme sentado/a |
| 62. Realizo las tareas sin cuidar demasiado los detalles |
| 63. Me enfrento con las personas que representan autoridad para mi |
| 64. He mentido por dinero |
| 65. Contesto antes de que terminen de preguntarme |
| 66. Me meto en peleas con facilidad |
| 67. Me involucro en actividades peligrosas sin considerar las consecuencias a largo plazo |
| 68. He sido irresponsable respecto a mis obligaciones (estudio, trabajo, familia…) |
| 69. Me resulta indiferente las necesidades de otras personas |
| 70. Me gusta seducir a los demás para que se fijen en mí |
| 71. Pienso que soy una persona muy influyente |
| 72. Si es necesario, manipulo sutilmente las situaciones para obtener lo que quiero |
| 73. Dudo de la lealtad de mis amigos/as |
| 74. Cuando alguien me enfada, exploto |
| 75. Me cuesta prestar atención cuando me hablan |
| 76. Me levanto de mi asiento constantemente |
| 77. Realizo las actividades de una forma descuidada |
| 78. Me disperso con facilidad mientras me hablan |
| 79. No acepto lo que me imponen las autoridades |
| 80. He engañado a otras personas para mi beneficio |
| 81. Respondo sin esperar que terminen de hablar |
| 82. En medio de peleas, he lanzado objetos a otras personas |
| 83. Asumo riesgos diarios por ser impulsivo/a |
| 84. Incumplo con mis obligaciones |
| 85. No me preocupan los sentimientos de los demás |
| 86. Me gusta llamar la atención con mi apariencia física |
| 87. Me gusta relacionarme con líderes |
| 88. Aunque les supongan inconvenientes, las personas de mi alrededor deberían atender mis peticiones |
| 89. Pienso que si cuento mis debilidades utilizarán la información para dañarme |
| 90. Tengo dificultades para controlarme cuando me enfado |
| 91. Me distraigo con tanta facilidad que no termino las cosas que comienzo |
| 92. Tengo dificultades para estar quieto/a |
| 93. No busco ser impecable en mis tareas |
| 94. Dejo las tareas sin terminar porque me distraigo |
| 95. Rechazo las peticiones de mis figuras de autoridad |
| 96. Si es necesario, no me importa mentir a otras personas |
| 97. Me cuesta esperar mi turno |
| 98. Cuando discuto puedo ejercer violencia física sobre otros |
| 99. Me comprometo a cosas que posteriormente me es difícil cumplir |
| 100. Me desespera escuchar los problemas de los demás |
| 101. Me gusta atraer la atención por mi apariencia física |
| 102. Me gusta relacionarme sólo con personas poderosas |
| 103. Pienso que si quieres conseguir lo que quieres, tienes que controlar a los demás |
| 104. Prefiero que mis amigos/as no sepan mucho de mí, por miedo a que lo usen en mi contra |
| 105. Pierdo el control de mis acciones cuando siento ira |
| 106. Me cuesta organizar el tiempo que le dedico a mis tareas |
| 107. Aunque moleste, no puedo quedarme quieto/a |
| 108. Por acabar pronto mis tareas, descuido que estén bien hechas |
| 109. Para mí es difícil gestionar el tiempo dedicado a hacer las tareas |
| 110. Rechazo las normas sociales |
| 111. Me he inventado acontecimientos por beneficio propio |
| 112. Aunque me lo proponga, no puedo esperar mi turno |
| 113. Puedo llegar a mostrarme agresivo/a con mis familiares |
| 114. No he cumplido con mis promesas |
| 115. No me afecta que mi conducta pueda ser dañina para alguien |
| 116. Causo vergüenza a mis amigos/as por ser dramático/a |
| 117. Tengo derecho a un trato privilegiado |
| 118. Pienso que detrás de los cumplidos hay motivos ocultos |
| 119. Me cuestan las actividades que requieren mucha concentración |
| 120. Me cuesta estar tranquilo/a en mi tiempo de ocio |
| 121. Me meto en conversaciones ajenas |
| 122. Me gusta captar la atención siendo exagerado/a en público |
| 123. En cualquier situación, espero que se me dé un trato preferente |
| 124. La amabilidad de la gente me genera desconfianza |
| 125. Me cuestan las tareas que requieren mucha atención |
| 126. Me cuesta permanecer quieto/a en mis actividades recreativas |
| 127. Me entrometo en asuntos de otras personas |
| 128. Me encanta que mis amigos/as me presten atención continuamente |
| 129. Me envidian por mis logros |
| 130. Desconfío de mi pareja |
| 131. Pierdo el material que necesito para mis actividades |
| 132. No puedo parar de moverme |
| 133. Me gusta ser el centro de atención allá donde vaya |
| 134. Causo envidia a otras personas |
| 135. Me gusta estar atento/a en mi relación de pareja para que no me traicionen |
| 136. No sé dónde pongo los materiales necesarios para mis actividades |
| 137. Siento la necesidad de estar continuamente en movimiento |
| 138. A los demás les iría mejor si siguiesen mis recomendaciones |
| 139. Me distraigo con cualquier ruido |
| 140. No puedo dejar de hablar, aunque moleste a otras personas |
| 141. Soy olvidadizo/a en las actividades cotidianas |
| 142. Los demás tienen que hacerme caso para que les vaya mejor |
| 143. Me distraigo con cualquier cosa |
| 144. Aunque tenga que estar en silencio, me es difícil estar callado/a |

Scoring Procedure:

1. **Facet/Trait scores (Dimensional)**

Facet/trait scores are calculated as the mean of the corresponding items.

Rule/law violations: (21 + 48 + 63 + 79 + 95 + 110) / 6 =

Impulsivity: (23 + 50 + 65 + 81 + 97 + 112 + 121 + 127) / 8 =

Physical aggression: (24 + 51 + 66 + 82 + 98 + 113) / 6 =

Risk-taking: (25 + 52 + 67 + 83) / 4 =

Irresponsibility: (26 + 53 + 68 + 84 + 99 + 114) / 6 =

Inattention: (17 + 20 + 44 + 47 + 60 + 75 + 78 + 91 + 94 + 106 + 109 + 119 + 125 + 131 + 136 + 139 + 141 + 143) / 18 =

Hyperactivity: (18 + 45 + 61 + 76 + 92 + 107 + 120 + 126 + 132 + 137 + 140 + 144) / 12 =

Lack of Rigid Perfectionsim: (19 + 46 + 62 + 77 + 93 + 108) / 6 =

Deceitfulness: (22 + 49 + 64 + 80 + 96 + 111) = 6

Lack of empathy: (27 + 54 + 69 + 85 + 100 + 115) / 6 =

Attention-Seeking: (1 + 28 + 55 + 70 + 86 + 101 + 116 + 122 + 128 + 133) / 10 =

Grandiosity: (6 + 33 + 56 + 71 + 87 + 102 + 117 + 123 + 129 + 134 + 138 + 142) / 12 =

Exploitative: (7 + 34 + 57 + 72 + 88 + 103) / 6 =

Suspiciousness: (8 + 35 + 58 + 73 + 89 + 104 + 118 + 124 + 130 + 135) / 10 =

Hostility: (9 + 36 + 59 + 74 + 90 + 105) / 6 =

1. **Disorder Scores (Categorical: Absence/Presence)**

1) Sum the two items corresponding to each diagnostic criterion (e.g., ***C1*** = items 21+48);

2) Check the cutoff value for each criterion (see Table 7)

3) Assign scores: “1” if the mean is equal to or higher than the cutoff (“0” if the score is lower).

4) Sum the criterion scores for each disorder and determine diagnostic status according to DSM-5 criteria

**Diagnostic Criteria by Disorder**

Antisocial: ***C1***: 21, 48; ***C2***: 22, 49; **C3**: 23, 50; **C4**: 24, 51; **C5**: 25, 52; **C6**: 26, 53; **C7**: 27, 54

Histrionic: ***C1***: 1, 28; ***C2***: 55, 70; ***C3***: 2, 29; 8 ***C4***: 86, 101; ***C5***: 3, 30; ***C6***: 116, 122; ***C7***: 4, 31; ***C8***: 5, 32

Narcissistic: ***C1***: 6, 33; ***C2***: 56, 71; ***C3***: 87, 102; ***C4***: 128, 133; ***C5***: 117, 123; ***C6***: 7, 34; ***C7***: 69, 85; ***C8***: 129, 134; ***C9***: 138, 142

Paranoid: ***C1***: 8, 35; ***C2***: 58, 73; ***C3***: 89, 104; ***C4***: 118, 124; ***C5***: 9, 36; ***C6***: 59, 74; ***C7***: 130, 135

Borderline: ***C1***: 10, 37; ***C2***: 11, 38; ***C3***: 13, 40; ***C4***: 67, 83; ***C5***: 14, 41; ***C6***: 12, 39; ***C7***: 15, 42; ***C8***: 90, 105; ***C9***: 16, 43

Inattention: ***C1***: 17, 20; ***C2***: 44, 47; ***C3***: 75, 78; ***C4***: 91, 94; ***C5***: 106, 109; ***C6***: 119, 125; ***C7***: 131, 136; ***C8***: 139, 143; ***C9***: 60, 141

Hyperactivity/Impulsivity: ***C1***: 18, 45; ***C2***: 61, 76; ***C3***: 92, 107; ***C4***: 120, 126; ***C5***: 132, 137; ***C6***: 140, 144; ***C7***: 65, 81; ***C8***: 97, 112; ***C9***: 121, 127

Supplementary Table 3**.** Table of specifications for the SEPBA

|  | **Personality Disorders and ADHD – DSM-5 Section II** | | | | | |  |  |
| --- | --- | --- | --- | --- | --- | --- | --- | --- |
| **Facets / Dimensional traits** | *Antisocial* | *Narcissistic* | *Paranoid* | *Borderline* | *Histrionic* | *ADHD* | **Other items** | ***Number of items (facet/trait)*** |
| *Rule/law violations* | (21/48)1 |  |  |  |  |  | 63,  79, 95, 110 | 6 |
| *Impulsivity* | (23/50)3 |  |  |  |  | (65/81)g (97/112)h (121/127)i |  | 8 |
| *Risk-taking* | (25/52)5 |  |  | (67/83)4 |  |  |  | 4 |
| *Irresponsibility* | (26/53)6 |  |  |  |  |  | 68,  84, 99, 114 | 6 |
| *Inattention* |  |  |  |  |  | (17/20)a (44/47)b (75/78)c (91/94)d  (106/109)e (119/125)f (131/136)g  (139/143)h (60/141)i |  | 18 |
| *Hyperactivity* |  |  |  |  |  | (18/45)a (61/76)b (92/107)c (120/126)d (132/137)e (140/144)f |  | 12 |
| *(Lack of) Rigid perfectionism* |  |  |  |  |  |  | 19,  46, 62, 77, 93, 108 | 6 |
|  |  |  |  |  |  |  |  |  |
| *Deceitfulness* | (22/49)2 |  |  |  |  |  | 64,  80, 96, 111 | 6 |
| *(Lack of) empathy* | (27/54)7 | (69/85)7 |  |  |  |  | 100,  115 | 6 |
| *Grandiosity* |  | (6/33)1 (56/71)2 (87/102)3  (117/123)5 (129/134)8  (138/142)9 |  |  |  |  |  | 12 |
| *Attention seeking* |  | (128/133)4 |  |  | (1/28)1 (55/70)2 (86/101)4 116/122)6 |  |  | 10 |
| *Exploitation* |  | (7/34)6 |  |  |  |  | 57,  72, 88, 103 | 6 |
| *Suspiciousness* |  |  | (8/35)1 (58/73)2 (89/104)3 (118/124)4 (130/135)7 |  |  |  |  | 10 |
| *Hostility* |  |  | (9/36)5 (59/74)6 | (90/105)8 |  |  |  | 6 |
| *Aggression (physical)* | (24/51)4 |  |  |  |  |  | 66,  82, 98, 113 | 6 |
| ***Number of externalizing items per disorder*** | 14 | 18 | 14 | 4 | 8 | 36 |  | 122 |
| *Items for internalizing facets/ traits* |  |  |  | *(10/37)1 (11/38)2 (13/40)3 (14/41)5 (12/39)6 (15/42)7 (16/43)9* | *(2/29)3 (3/30)5 (4/31)7 (5/32)8* |  | 28  *22* |  |
| **Total number of items per disorder** | 14 | 18 | 14 | 18 | 16 | 36 |  | 116______ |
| 116 (items of disorders) + 28 (other items for facets/traits) = 122 (externalizing items) + *22* (internalizing items) = **144 SEPBA items** | | | | | | | | |

Note: This table shows the correspondence between SEPBA facets/traits and diagnostic criteria for personality disorders and ADHD (*DSM-5*, Section II). Rows list facets/traits (e.g., impulsivity, grandiosity), and columns list disorders. Each cell includes SEPBA item pairs (e.g., *(21/48)*) and the DSM-5 diagnostic criterion they assess (e.g., *(21/48)1* indicates that items 21 and 48 measure criterion 1 of Antisocial Personality Disorder). Item numbers listed under *Other items* refer to items used to measure facets/traits but not diagnostic criteria. The final column summarizes items per facet, and the final rows report the total item counts (144), distinguishing externalizing (122) and internalizing (22) items.

Supplementary Table 4. Instruments used to provide convergent and discriminant evidence for the facets/traits and disorders assessed by the SEPBA

|  | Cronbach’s α α | McDonald’s  ω |
| --- | --- | --- |
| International Personality Disorder Examination Screening Questionnaire (IPDEQ) | | |
| Borderline disorder | .70 | .70 |
| Histrionic disorder | .50 | .47 |
| Antisocial Disorder | .65 | .66 |
| Narcissistic disorder | .48 | .55 |
| Paranoid disorder | .60 | .61 |
| Spanish version of the Personality Inventory for DSM-5 — Short Form (PID-5-SF) | | |
| Attention seeking | .91 | .91 |
| Callousness | .83 | .83 |
| Deceitfulness | .81 | .81 |
| Hostility | .80 | .82 |
| Grandiosity | .77 | .77 |
| Manipulativeness | .81 | .81 |
| Distractibility | .87 | .87 |
| Impulsivity | .91 | .91 |
| Irresponsibility | .69 | .66 |
| (Lack of) rigid perfectionism | .83 | .83 |
| Risk taking | .92 | .92 |
| Suspiciousness | .74 | .75 |
| Adult ADH Self-Report Scale (ASRS v1.1) | | |
| Section A | .75 | .73 |
| Section B | .88 | .87 |
| Inattention | .78 | .78 |
| Impulsivity | .74 | .73 |
| Externalizing Spectrum Inventory (ESI) | | |
| Rebelliousness | .84 | .84 |
| Physical Aggression | .88 | .88 |
